# Aggressive prostate cancer is associated with pericyte dysfunction

**DOI:** 10.1002/1878-0261.70135

**Published:** 2025-10-21

**Authors:** Anabel Martinez‐Romero, Ane Martinez‐Larrinaga, Joaquim Grego‐Bessa, Saioa Garcia‐Longarte, Hielke van Splunder, Ianire Astobiza, Amaia Ercilla, Laura Bozal‐Basterra, Isabel Mendizabal, Pilar Villacampa, Arkaitz Carracedo, Mariona Graupera

**Affiliations:** ^1^ Endothelial Pathobiology and Microenvironment Group Josep Carreras Leukaemia Research Institute (IJC) Barcelona Catalonia Spain; ^2^ Department of Physiological Sciences, Faculty of Medicine and Health Sciences University of Barcelona Spain; ^3^ Center for Cooperative Research in Biosciences (CIC bioGUNE), Basque Research and Technology Alliance (BRTA) Derio Spain; ^4^ CIBERONC, Instituto de Salud Carlos III Madrid Spain; ^5^ Advanced Microscopy Unit. Institut de Recerca Contra la Leucèmia Josep Carreras (IJC), ICO‐Hospital Germans Trias i Pujol Universitat Autònoma de Barcelona Spain; ^6^ Traslational Prostate Cancer Research Lab CIC bioGUNE‐Basurto, Biocruces Bizkaia Health Research Institute Bilbao Spain; ^7^ Ikerbasque; Basque Foundation for Science Spain; ^8^ Biochemistry and Molecular Biology Department University of the Basque Country (UPV/EHU) Bilbao Spain; ^9^ ICREA, Institució Catalana de Recerca i Estudis Avançats, Pg. Lluís Companys 23 Barcelona Spain

**Keywords:** pericytes, prostate cancer, TGFβ signaling, vessels

## Abstract

Pericytes are intrinsic components of vessels that regulate vascular stability and permeability. Aberrant vascularization is a hallmark of cancer, although the contribution of pericytes to this process is poorly understood. Here, we undertook a combined computational and experimental approach to identify the molecular reprogramming of prostate pericytes during cancer pathogenesis and progression. Analysis of human prostate cancer and murine models showed that prostate tumors exhibit a disequilibrium between endothelial and pericyte content with prognostic potential. Deeper molecular analysis revealed that there is no overt loss of pericytes in prostate tumors but rather a dysfunction that is concomitant with altered expression of a subset of cellular markers. We translate this finding into a simplified signature that discriminates pericyte abundance versus function. Leveraging single‐cell RNA sequencing data, we find that TGF‐β governs the molecular changes that underlie pericyte dysfunction in tumors. This mechanism is associated with reduced expression of contractility markers, enlargement of the vascular lumen, and increased permeability in prostate cancer. This study revisits the paradigm of the reduced number of pericytes in favor of their dysfunction in tumors and the importance of paracrine signaling in this process.

## Graphical abstract

Tumor‐produced TGF‐β drives pericyte dysfunction in prostate cancer. This dysfunction is characterized by downregulation of some canonical pericyte markers (i.e., DES, CSPG4, and ACTA2) while maintaining the expression of others (i.e., PDGFRB, NOTCH3, and RGS5). This transcriptional shift is associated with reduced vascular contractility and increased vessel permeability, leading to extravasation and tumor dissemination.

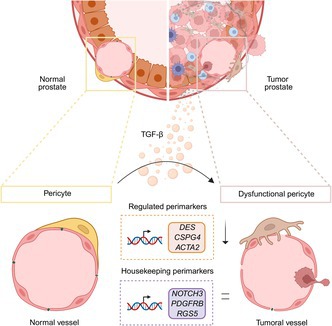

**Abbreviations**
M: metastasis
MYH11: myosin heavy chain 11
N: normal tissue
Pb‐Cre4: androgen‐dependent ARR_2_B probasin promoter
PT: primary tumor
scRNAseq: single‐cell RNA sequencing
TBST: TBS buffer with 0.1% Tween 20
TCGA: the cancer genome atlas program
TGF‐β: transforming growth factor‐β
UMAP: Uniform Manifold Approximation and Projection
vSMC: vascular smooth muscle cells
αSMA: α smooth muscle actin

## 1 Introduction

Cancers are complex ecosystems in which tumor cells intermingle with noncancerous counterparts and matrix components, collectively known as the tumor microenvironment [1, 2, 3]. A subset of these noncancerous cells, such as fibroblasts, endothelial cells, and mural cells, is reprogrammed versions of normal stromal components [2], and their relevance in tumor evolution is now well‐documented. Yet, how the stromal compartment regulates tumorigenesis, from onset to progression, is not fully understood.

Prostate cancer is the most common cancer type in men, and its prevalence and socioeconomic impact are rising [4]. Histological and molecular characteristics of prostate cancer can inform about disease aggressiveness [5], and these parameters do not solely rely on tumor cell‐intrinsic features. Indeed, the tumor microenvironment also plays an important role in prostate tumorigenesis [6, 7, 8], with mesenchymal cells emerging as significant modulators of disease progression [8]. Mesenchymal cells include both fibroblasts and mural cells, yet very little is known about the contribution of the latter to prostate cancer initiation and progression.

Mural cells comprise both pericytes and vascular smooth muscle cells (vSMC). Pericytes are found throughout the body, covering capillaries and embedded within the basement membrane [9, 10]. Instead, vSMC refers to mural cells covering large arteries and veins that are separated from endothelial cells by a layer of fibroblasts and extracellular matrix. The main function of mural cells is to provide structural support to blood vessels and maintain organ homeostasis by promoting vascular stability and regulating vascular permeability [9, 10]. Yet, the identification of pericytes versus vSMC is not always easy, as the transition from one to the other is progressive. This is even more challenging in tumors where the vasculature grows fast and in a highly aberrant manner [1]. Hence, in our study, we will refer to the term pericytes when studying tumor mural cells. Due to their integral part in blood vessels, pericytes have been postulated to play an active role in tumor angiogenesis. Tumor vessels are often devoid of pericytes [11, 12, 13], and low pericyte coverage correlates with poor patient prognosis [14, 15]. Also, reduced pericyte coverage favors metastasis [16]. However, we lack a detailed understanding of the causes and consequences of this process.

The transforming growth factor‐β (TGF‐β) family comprises a large group of pleiotropic cytokines involved in development, tissue homeostasis, and pathological processes [17, 18]. TGF‐β signaling regulates a variety of cell functions, including proliferation, differentiation, and migration. From a physiological standpoint, TGF‐β signaling nurtures embryonic development, angiogenesis, wound healing, and immune responses [17, 18]. Aberrant TGF‐β signaling disrupts immune tolerance, promotes inflammation, and underlies the pathogenesis of fibrosis and cancer [19]. This cytokine is also involved in cancer‐promoting processes of stromal cells [8, 20].

Here, we show that pericyte dysfunction in prostate cancer is a previously unidentified process that accompanies the acquisition of metastatic capacity. Importantly, this process is associated with altered permeability of vessels, which is proposed to facilitate the dissemination of cancer cells. We identify that pericyte dysfunction is orchestrated by TGF‐β signaling.

## 2 Materials and methods

### 2.1 Mouse models

All experiments were performed in agreement with the guidelines and legislation of the Basque Ministry of Agriculture, Livestock, Fisheries and Food (Basque Country, Spain), following protocols approved by the local Ethics Committees of the Biosafety and Animal Welfare Committee at CIC bioGUNE in concordance with the recommendation of AAALAC (approved protocol P‐CBG‐CBBA‐0121) as described [21]. Mice were maintained in controlled environmental conditions, with time‐controlled lighting on standard 12 : 12 light:dark cycles, 30–50% of humidity, and controlled temperature at 22 ± 2 °C. Diet and water were provided *ad libitum*. 4‐ and 6‐month‐old male mice were used for our studies. Mice were fasted for 6 h prior to tissue harvest to prevent metabolic alterations due to immediate food intake. At the experimental endpoint, all mice were sacrificed by CO_2_ inhalation followed by cervical dislocation. The Pten loxP and Lkb1 loxP conditional knockout alleles have been described elsewhere [21, 22, 23]. Briefly, the conditional tissue‐specific Pten knockout (C57/BL6/129sv; Pb‐Cre4; Pten lox/lox) model was provided by Pier Paolo Pandolfi (Beth Israel Deaconess Cancer Center, Boston, MA, USA). The conditional tissue‐specific Lkb1 null homogeneous background model (FVB; Lkb1lox/lox) was from Mouse Models of Human Cancer Consortium in a pure FVB background. The Lkb1 loxP mouse was backcrossed into C57BL/6J for four generations to obtain animals with a genetic background enriched in C57/BL6 (> 90% C57/BL6). Next, we crossed Lkb1 lox/lox mice with Pb‐Cre4 and Pten lox/lox mice for at least four generations to obtain a founder colony with a mixed homogeneous background, and experimental animals were generated. Littermates were analyzed when possible. However, due to the complexity of the allele combination, the different genotypes of interest with Pten and/or Lkb1 alteration were obtained from parallel contemporary breeding pairs. Probasin Cre was always retained in male mice, since in females, Pb‐Cre4 expression in utero can lead to recombination in embryos during pregnancy. The Cre recombinase expression, under the control of androgen‐dependent ARR2B probasin promoter (Pb‐Cre4), allowed the deletion of Pten in the prostate epithelium at puberty. Prostate *Pten/Lkb1*‐deleted male mice were termed pc−/+ (heterozygous) or pc−/− (homozygous knockout) for each gene. Prostate sample collection from the *Pten* knockout model was performed at 6 months of age, and the prostate from the *Pten/Lkb1*‐deleted male was performed at 4 months.

### 2.2 Immunofluorescence

All immunostainings were performed in 5‐μm‐thick paraffin sections. Briefly, antigen retrieval was performed with citrate buffer at pH = 6 and permeabilization with PBS + 0.2% Tween 20; primary antibodies were incubated overnight at 4 °C: Phospho‐S6 Ribosomal Protein (Ser235/236; Cell Signaling cat. no. 4857 diluted 1 : 100, Danvers, MA, USA), CD31 (Abcam, ab28364 diluted 1 : 50; Proteintech, cat. no. 28083 diluted 1 : 200; BD Pharmingen, cat. no. 550274 diluted 1 : 100, Cambridge, UK), Desmin (AF3844‐SP, diluted 1 : 100), Notch3 (Abcam, ab23426, diluted 1 : 100; R&D System, Minneapolis, MN, USA), αSMA (cat. no. C6198 diluted 1 : 300; Sigma‐Aldrich, Saint Louis, MI, USA), MYH11 (ab224804, diluted 1 : 50; Abcam), Ter119 (MAB1125, diluted 1 : 50; R&D System). Secondary antibodies were incubated for 2 h at room temperature (Fluorescent coupled antibodies: Life Technologies A31573, Invitrogen A11008, A21208, and A48269 diluted 1 : 400; IMMPRESS HRP anti‐Rabbit, Vector MP‐7451, and anti‐Rat‐HRP, DAKO P0450 diluted 1 : 100, Carlsbad, CA, USA). Alexa Fluor 594 Tyramide SuperBoost Kit (B40925; Invitrogen) was used for double IF, and ImmPACT® DAB Substrate Kit (Vector, SK‐4105) for pS6 detection. DAPI was used for nuclei counterstaining (S33025 diluted 1 : 5000; Invitrogen). Samples were mounted with Shandon™ Immu‐Mount™ or Fluoromount‐G (SoutherBiotech, 0100‐01, Birmingham, AL, USA).

### 2.3 Imaging and quantification

Imaging and quantification analyses were performed as described [24]. Briefly, we used a Leica TCS SP5 confocal microscope and a Leica Stellaris 8, in combination with Adobe Photoshop 2022 and ImageJ software for image editing and quantification, respectively. Images were taken from five independent vessel areas in each mouse, and at least five mice per genotype were analyzed as previously reported [25]. The vessel area was measured using the CD31‐positive area, which was quantified by dividing the percentage of CD31‐positive area by the total tissue area. The coverage vessel area was measured using Desmin, MYH11, or αSMA and CD31 channels. First, a manual threshold was set to obtain the CD31‐positive area, which served as a CD31 mask and defined the coverage region. Then, using the defined CD31 mask, the integrated Desmin, MYH11, or αSMA expression was measured only in the CD31 mask. Erythrocyte cell area was determined by using the Ter119‐positive area outside the vessels, which was quantified by dividing the percentage of Ter119‐positive area by the total tissue area.

### 2.4 Cell culture

Brain pericytes were isolated as described [26, 27]. Briefly, brains were homogenized with a blade and incubated in a papain enzymatic solution (Worthington, LK003150). After several rounds of centrifugation, cells were resuspended in EGM2 complete medium and plated on 0.02% collagen I‐coated plates (354249; BD Biosciences, Franklin Lakes, NY, USA). Cells were cultured at 37 °C in a 5% CO2 atmosphere in EGM‐2 supplemented with EGM‐2 BulletKit (#CC‐3162; Lonza, Basel, Switzerland), 20% FBS, and 1% penicillin/streptomycin. The medium was changed every 3 days until the cells reached confluency, followed by a passage into a new well to expand the culture. After the third passage, cells were maintained and propagated in pericyte medium composed of a basal medium supplemented with growth factors (1252; ScienCell, Carsbad, CA, USA), 2% FBS, and 1% penicillin/streptomycin. Pericytes were characterized after passage 6. For pharmacological treatment and inhibition of TGF‐β, pericytes were cultured for 24 h followed by treatment with vehicle, 5 μg mL^−1^ TGF‐β1 (167100‐21‐B; Prepotech, Cranbury, NY, USA), and/or 3 μmol l^−1^ SIS3 (SMAD3 inhibitor) for 24 or 48 h.

### 2.5 Protein extraction and immunoblotting

Cells were lysed in ice‐cold lysis buffer [RIPA (20‐188; Millipore, Burlington, MA, USA) supplemented with MgCl_2_ and Benzonase (E1014‐5KU; Millipore)] for 10 min and then centrifuged at > 16 000 ***g*** for 15 min at 4 °C. Protein concentrations of the supernatants were measured with the Pierce BCA Protein Assay Kit (23225; Thermo Fisher Scientific, Waltham, MA, USA) as described [24]. Total cell lysates were resolved on 4–12% Bis‐Tris polyacrylamide gels (WG1403BOX; Invitrogen) and transferred onto nitrocellulose membranes (66485; Pall Corporation, Port Washington, NY, USA) for 1 : 30 h at 100 V. The membranes were blocked in 5% milk in TBST (TBS buffer with 0.1% Tween 20) for 1 h and then incubated overnight with the following specific primary antibodies diluted in 0.002% azida TBS‐T: anti‐pSMAD3 (9520; diluted 1 : 1000; Cell Signaling), anti‐Desmin (ab15200; diluted 1 : 1000; Abcam), anti–β‐actin (A5441; diluted 1 : 20 000; Sigma‐Aldrich), anti‐CD13 (AF2335; diluted 1 : 1000; R&D), anti‐NG2 (AB5320; diluted 1 : 1000; Millipore), anti‐αSMA (14968; diluted 1 : 1000; Cell Signaling), and anti‐vinculin (V9131; diluted 1 : 2000; Sigma). After three washes in TBST, the membranes were incubated with the appropriate horseradish peroxidase–conjugated secondary antibody, washed five times in TBST, and developed with reagents for enhanced chemiluminescence. The following secondary antibodies from Promega (Madison, WI, USA) were used (all diluted 1 : 5000): goat anti‐rabbit (W401B) and goat anti‐mouse (W4021). All immunoblots were performed at least three times independently, and representative experiments were included in the figures. Quantifications were done using ImageJ.

### 2.6 RNA extraction, cDNA synthesis, and quantitative polymerase chain reaction

RNA was extracted using Maxwell^®^ RSC simplyRNA cell kit (AS1390; Promega). Reverse transcription was performed from 1000 ng of RNA using the High‐Capacity cDNA Reverse Transcription Kit. For quantitative polymerase chain reaction, a LightCycler 480 System was used with LightCycler 480 SYBR Green I Master kit and specific primers. mL32 was used as a housekeeping gene.

### 2.7 Bulk RNA sequencing analysis of mouse pericytes

Four independent primary pericyte isolates were treated with vehicle and TGF‐β1 (5 μg mL^−1^). For the RNA‐seq data processing, reads were aligned to the mouse reference genome (mm10) using STAR (version_2.7.5c) in two‐pass mode following STAR best practices and recommendations. The quality of the data was evaluated using STAR (v 2.7.5c) and samtools (version 1.15). PCR duplicates were removed from aligned bam files using samtools (version 1.15). Read counts were extracted from the aligned bam files using subread's FeatureCounts (v 2.0.3). For differential gene expression analysis, the raw count matrix was imported into R and analyzed using the DESeq2 package (v1.34.0) [28]. A *DESeqDataSet* object was first constructed to encapsulate the count data and associated experimental metadata. The analysis followed the standard DESeq2 workflow, which includes normalization using the *DESeq()* function, estimation of dispersion, and fitting of a generalized linear model to each gene to compute log fold changes and statistical significance. We did not apply an explicit prefiltering threshold based on *counts* or *baseMean* values prior to modeling. Instead, we relied on DESeq2's internal independent filtering step implemented in the *results ()* function. This data‐adaptive filtering automatically removes genes with low mean normalized counts that are unlikely to reach statistical significance, thereby increasing detection power while controlling the false discovery rate. As clarified by the DESeq2 authors, this approach is consistent with best practices and avoids the need for manual filtering. Statistical significance of differential expression was assessed using Wald tests, with the results corrected for multiple testing using the Benjamini‐Hochberg procedure. A threshold of |log_2_FC >1.5| and FDR adjusted *P*‐value < 0.05 was applied to identify differentially expressed genes. For enrichment functional analysis, we used a ClusterProfiler package (v4.2.2) in R. The *enrichGO* function was utilized to identify enriched Gene Ontology terms, using the categories for Biological Process. Moreover, the *enrichKEGG* function was applied to detect enriched pathways in the Kyoto Encyclopedia of Genes and Genomes (KEGG) database. Enrichment analyses were performed by preparing a gene list with associated statistical measures and executing the enrichment functions with appropriate parameters, such as organism database and *P*‐value cutoffs.

### 2.8 Single‐cell RNA‐seq data and analysis

Publicly available single‐cell RNA‐Seq (scRNAseq) datasets of primary prostate cancer from 58 patients were obtained from several studies [29, 30, 31] (summary of patients' data can be found in Table S1). All downstream preprocessing was performed using Seurat (v4.1.0). We aggregated individual datasets using the *merge* function in Seurat. As part of our data analysis, we removed genes with no reads and low‐quality cells. Specifically, we excluded cells with: (i) fewer than 250 total genes, (ii) mitochondrial transcript fraction greater than 20%, and (iii) a complexity index greater than 0.8. Additionally, genes detected in five or fewer cells were excluded. Our quality control criteria were based on protocols from https://hbctraining.github.io/scRNA‐seq/lessons/04_SC_quality_control.html and the Tonsil Atlas [32]. To estimate doublets, which are pairs of cells sequenced under the same cellular barcode, typically captured in the same droplet, we used the DoubletFinder package in R. This tool simulates artificial doublets using existing data and compares them with actual cells to identify potential doublets. Finally, to ensure the representation of rare cell types across individuals and maximize the effective capture of cellular diversity, we filtered out patients with fewer than 1000 cells [33, 34]. Rare cell types often cluster with more common cell types and can only be reliably detected when tens of thousands of cells from multiple patient biopsies are profiled together. This threshold also minimizes biases introduced by low‐cell‐count datasets, which may lack the statistical power to identify less abundant populations or exhibit disproportionate variability due to stochastic sampling effects. For normalization, we applied the *NormalizeData* function with the LogNormalize method and a scale factor of 10 000. This process involves dividing the raw gene counts of each cell by the total counts for that cell, multiplying by the scale factor, and then performing log‐normalization as log(1 + x). To select the number of highly variable genes (HVG) for further analysis, we first calculated the distribution of nonzero counts across all genes using the RNA assay data. This was achieved by applying the *colSums* function from the Matrix package (v 1.6.0). We then identified the number of HVG by adding 100 to the third quartile of this distribution, establishing a threshold for variability selection. Once the target number of HVGs was defined, we applied Seurat's *FindVariableFeatures* function with method = “vst” to identify the most variable genes according to standardized dispersion measures. Following HVG selection, we performed a *z*‐score transformation on the normalized values using Seurat's *ScaleData* with default parameters, followed by principal component analysis using *RunPCA*. To correct for batch effects, we utilized Harmony with patients' IDs as covariates, which has been demonstrated to scale well to hundreds of thousands of cells and ranks among the top integration tools [35]. Finally, clustering analysis was performed based on the edge weights between any two cells, utilizing a shared nearest‐neighbor graph produced by the Louvain algorithm, implemented in Seurat's *FindNeighbors* and *FindClusters* functions. The identified clusters were visualized using the Uniform Manifold Approximation and Projection (UMAP) method. For annotation purposes, we performed a “one‐vs‐all” differential expression analysis to identify markers specific to each cluster. This was done using the *FindAllMarkers* function with the default non‐parametric Wilcoxon rank‐sum test and Bonferroni correction for multiple comparisons. Lower clustering resolutions were used, as our primary aim was to identify general cell type populations. The marker genes used for population annotation were as follows: epithelial cells (*EPCAM*, *KRT8*, *KRT18*), macrophages (*CD68*, *C1QA*, *C1QB*), T cells (*CD8A*, *CD4*, *CD8B*), B cells (*CD79A*, *CD79B*, *CD27*), endothelial cells (*PECAM1*, *CDH5*, *VWF*), fibroblasts (*PDGFRA*, *COL1A1*, *DCN*), and pericytes (*PDGFRB*, *RGS5*, *ACTA2*). This pipeline was followed in various scenarios where marker estimation was required for population identification.

### 2.9 Ligand–receptor analysis

To identify cell‐to‐cell interactions within the scRNAseq dataset, we employed NicheNet [36]. This analysis leverages extensive public databases, including KEGG, ENCODE, and PhosphoSite, to identify downstream effectors such as transcription factors and targets of receptors. NicheNet analysis outputs three key matrices: the “*ligand‐target prior model*,” the “*ligand‐receptor network*,” and the “*weighted integrated networks*.” We utilized these tools on our cell‐type‐labeled whole object. Specifically, we designated the mural cell cluster as the receiver cell type and all other clusters as the sender cell types. The conditions of interest were “Tumor” versus “Normal,” as analyzed in the *nichenet_seuratobj_aggregate* function.

### 2.10 Statistics

Microscopy and immunoblotting data were analyzed with the GraphPad Prism software and are presented as mean ± SEM (error bars). The sample size for each experiment is indicated in the corresponding figure legend. All figures are displayed with individual data points that indicate biological replicates and with the SEM as error bars. At least three biological replicates were used. *P* values considered statistically significant were as follows: *P ≤ 0.05, **P ≤ 0.01, ***P ≤ 0.001, and ****P ≤ 0.0001.

## 3 Results

**Fig. 1:**
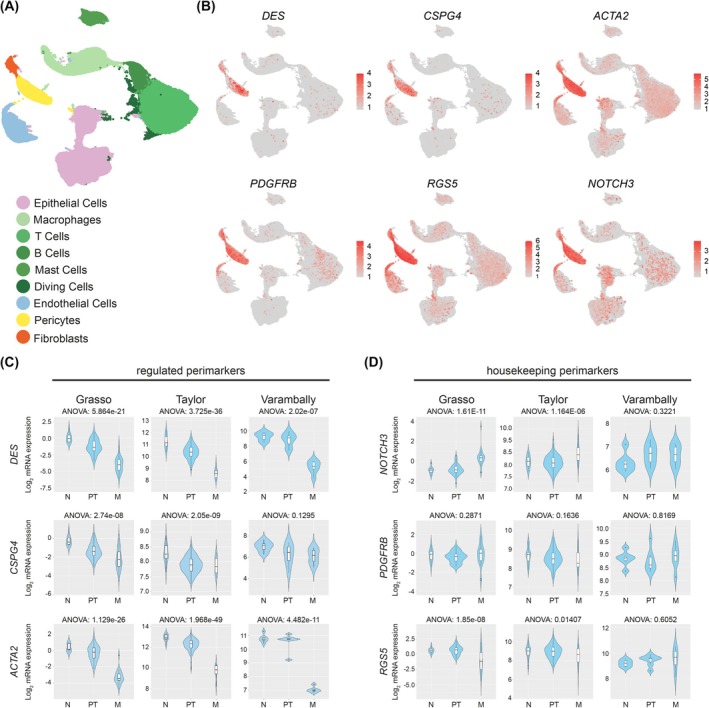
Identification of regulated and housekeeping perimarkers in human prostate data. (A) Uniform Manifold Approximation and Projection (UMAP) plot of the different cell populations found in human prostate data (detailed information on scRNAseq data sets is included in Table S1). (B) UMAP plots showing the expression of *DES, CSPGA, ACTA2, PDGFRB, RGS5*, and *NOTCH3* genes across all clusters. (C) Violin plots depicting the expression of *DES*, *CSPG4*, and *ACTA2* between non‐tumoral/normal (N), primary tumor (PT), and metastatic (M) PCa specimens in the indicated datasets. The *y*‐axis represents the log_2_‐normalized gene expression. Statistical analysis was performed by ANOVA. (D) Violin plots depicting the expression of *NOTCH3*, *PDGFRB*, and *RGS5* between non‐tumoral/normal (N), primary tumor (PT), and metastatic (M) PCa specimens in the indicated datasets. The *y*‐axis represents the log_2_‐normalized gene expression. Statistical analysis was performed by ANOVA.

The role of pericytes in prostate cancer is poorly understood, partially due to a lack of selective markers for pericytes in the prostate [9, 10]. Hence, we first proceeded with an in‐depth characterization of prostate‐specific pericytes to identify consensus cell markers using a compendium of scRNAseq studies in normal and neoplastic tissues [29, 30, 31] (Fig. 1A and Table S1). As represented using UMAP, we identified a series of markers whose expression was enriched in prostate pericytes (i.e., *DES, RGS5*, *NOTCH3, PDGFRB, ACTA2, CSPG4*), most of which are postulated as robust pericyte markers across tissues [10, 37] (Fig. 1B and Fig. S1A). Next, we leveraged these markers to characterize potential pericyte‐related vascular phenotypes in prostate neoplastic tissue. To this end, we employed bulk human transcriptomic datasets of various patient cohorts, including normal tissue (N), primary tumor (PT), and metastasis (M), and inferred the abundance of pericytes by applying the pericyte markers validated in Fig. 1B. Intriguingly, we observed that the selected genes clustered into two distinct behaviors. On the one hand, a set of markers exhibited a consistent reduction in their expression along prostate cancer progression (i.e., *DES, CSPG4*, and *ACTA2* (Ref [25, 38])) (Fig. 1C), which we labeled as “regulated perimarkers.” On the other hand, a second set of genes remained unperturbed in prostate cancer progression (i.e., *NOTCH3*, *PDGFRB*, and *RGS5*) (Fig. 1D), which we termed “housekeeping perimarkers.”

**Fig. 2:**
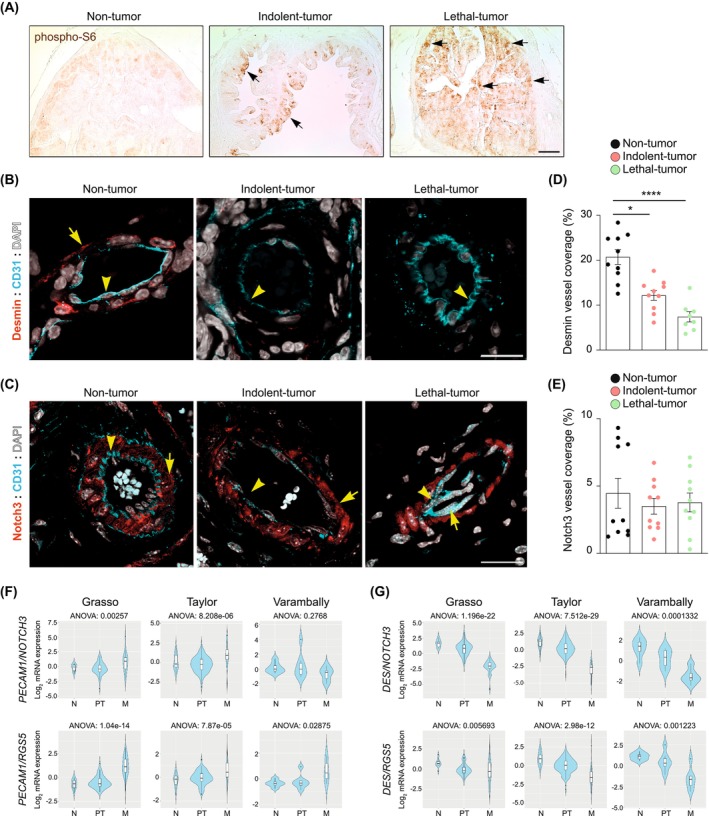
Prostate cancer progression is associated with a reduction in Desmin‐positive pericytes. (A) Representative images of non‐tumor (Pb‐Cre‐PTEN^WT/WT^), indolent tumor (Pb‐Cre‐PTEN^flox/flox^), and lethal tumor (Pb‐Cre‐PTEN^flox/WT^; LKB1^flox/flox^) prostates stained for phospho‐S6 (brown). Staining was performed in *n* = 3 mice per genotype. Scale bars, 25 μm. Arrows indicate epithelial cells positive for phospho‐S6. (B) Representative images of non‐tumor (Pb‐Cre‐PTEN^WT/WT^), indolent tumor (Pb‐Cre‐PTEN^flox/flox^), and lethal tumor (Pb‐Cre‐PTEN^flox/WT^; LKB1^flox/flox^) prostates stained for endothelial cells (CD31, cyan), pericytes (Desmin, red), and DAPI (white). Staining was performed in *n* = 10 Pb‐Cre‐PTEN^WT/WT^ and Pb‐Cre‐PTEN^flox/WT^; LKB1^flox/flox^ mice, and in *n* = 8 Pb‐Cre‐PTEN^flox/flox^ mice. Scale bars, 25 μm. Arrowheads point to the endothelial cells, and arrows show Desmin positivity. (C) Representative images of non‐tumor (Pb‐Cre‐PTEN^WT/WT^), indolent tumor (Pb‐Cre‐PTEN^flox/flox^), and lethal tumor (Pb‐Cre‐PTEN^flox/WT^; LKB1^flox/flox^) prostates stained for endothelial cells (CD31, cyan), pericytes (Notch3, red), and DAPI (white). Staining was performed in *n* = 10 mice per genotype. Scale bars, 25 μm. Arrowheads point to the endothelial cells, and arrows show Notch3 positivity. (D) Quantification of Desmin vessel coverage (%). *n* = 10 Pb‐Cre‐PTEN^WT/WT^ and Pb‐Cre‐PTEN^flox/WT^; LKB1^flox/flox^ mice, and *n* = 8 Pb‐Cre‐PTEN^flox/flox^ mice. Data represent mean ± SEM. Each dot corresponds to an individual mouse. Statistical analysis was performed by Kruskal–Wallis test. *P ≤ 0.05 and ****P ≤ 0.0001. (E) Quantification of Notch3 vessel coverage. *n* = 10 mice per genotype. Data represent mean ± SEM. Each dot corresponds to an individual mouse. Statistical analysis was performed by Kruskal–Wallis test. (F) Violin plots depicting the ratio expression of *PECAM1* and pericyte genes (*NOTCH3*, *RGS5*) showing progression [non‐tumoral/normal (N), primary tumor (PT), and metastatic (M) (prostate cancer, PCa)]. The *y*‐axis represents the log_2_‐normalized gene expression. Statistical analysis was performed by ANOVA. (G) Violin plots depicting the ratio expression of *DES* and pericyte genes (*NOTCH3*, *RGS5*) showing progression [non‐tumoral/normal (N), primary tumor (PT), and metastatic (M) (prostate cancer, PCa)]. The *y*‐axis represents the log_2_‐normalized gene expression. Statistical analysis was performed by ANOVA.

To explore the alterations underlying the vascular bed *in situ*, we utilized a series of mouse models with varying degrees of prostate pathology, including wild‐type prostate specimens, indolent tumors (prostate‐conditional *Pten* knockout, nonmetastatic [22, 23]), and metastatic prostate tumors (prostate‐conditional *Pten* heterozygous and *Stk11* knockout [21]). First, we stained tissues with phospho‐S6 staining as a proxy of signaling perturbations associated with tumor pathology and found increased levels of phospho‐S6 associated with tumor progression (Fig. 2A). Next, we monitored the same specimens with regulated (Desmin) and housekeeping (Notch3) perimarkers. Given that these markers also detect the tumor stroma around the glands, we used CD31 (*Pecam1*), an established vessel marker (Fig. 2B,C, Fig. S2A,B), to distinguish the perivascular region. To study pericyte biology *in situ*, we focused on perivascular Desmin and Notch3 staining, aided by CD31 immunoreactivity. We confirmed a robust reduction in Desmin immunostaining associated with disease aggressiveness (Fig. 2B,D). In contrast, the analysis of Notch3 staining corroborated the human data and exhibited retention of perivascular signal in prostate tumors (Fig. 2C,E). Additionally, we identified a positive correlation between NOTCH3 expression and key downstream targets using Taylor and the Cancer Genome Atlas (TCGA) program, suggesting the activation of the pathway in prostate cancer pericytes (Fig. S2C).

The distinct behavior of the two classes of pericyte markers during prostate cancer progression led us to propose a paradigm shift in the current understanding of cancer pericytes: that, in addition to the reported loss of pericytes in the tumor vasculature [14, 15], pericytes that are retained in intratumoral vessels exhibit functional alterations that could influence disease progression. The lack of robust markers might have acted as a confounding factor, leading to the interpretation that loss of immunoreactivity for proteins such as Desmin is indicative of pericyte loss. To revisit the current paradigm, we postulate that, similar to the physiological process of pericyte differentiation [9, 10, 39], a series of pericyte markers would serve as housekeeping genes for the presence and abundance of these cells (*NOTCH3, PDGFRB, RGS5*), whereas others would provide information about their functionality (*DES* and *CSPG4*). To translate this notion into quantifiable evidence, we undertook two complementary strategies. On the one hand, using the scRNAseq dataset, we observed a reduction in the normalized expression of *DES* and *CSPG4* in tumor pericytes, whereas no consistent trend was detected for *NOTCH3, PDGFRB*, or *RGS5* (Fig. S2D). On the other hand, we built gene expression scores to illustrate distinct vascular scenarios. First, a score for vascular mural cell coverage was calculated by inferring the ratio of *PECAM1* to *NOTCH3* or *RGS5* (*PECAM1/NOTCH3; PECAM1/RGS5*), where increased values would indicate an elevated endothelial to mural cell ratio, and hence, naked vessels. This analysis revealed increased naked vessels in 2 out of 3 datasets with specimens of normal prostate, primary tumor, and metastasis (Fig. 2F). Second, we built a score of mural cell functionality, based on the expression of *DES* versus *NOTCH3* or *RGS5* (*DES/NOTCH3*; *DES/RGS5*), where a drop in the score would be indicative of mural cell dysfunction. The analysis of various prostate cancer cohorts confirmed increased mural cell dysfunction in aggressive disease (Fig. 2G).

**Fig. 3:**
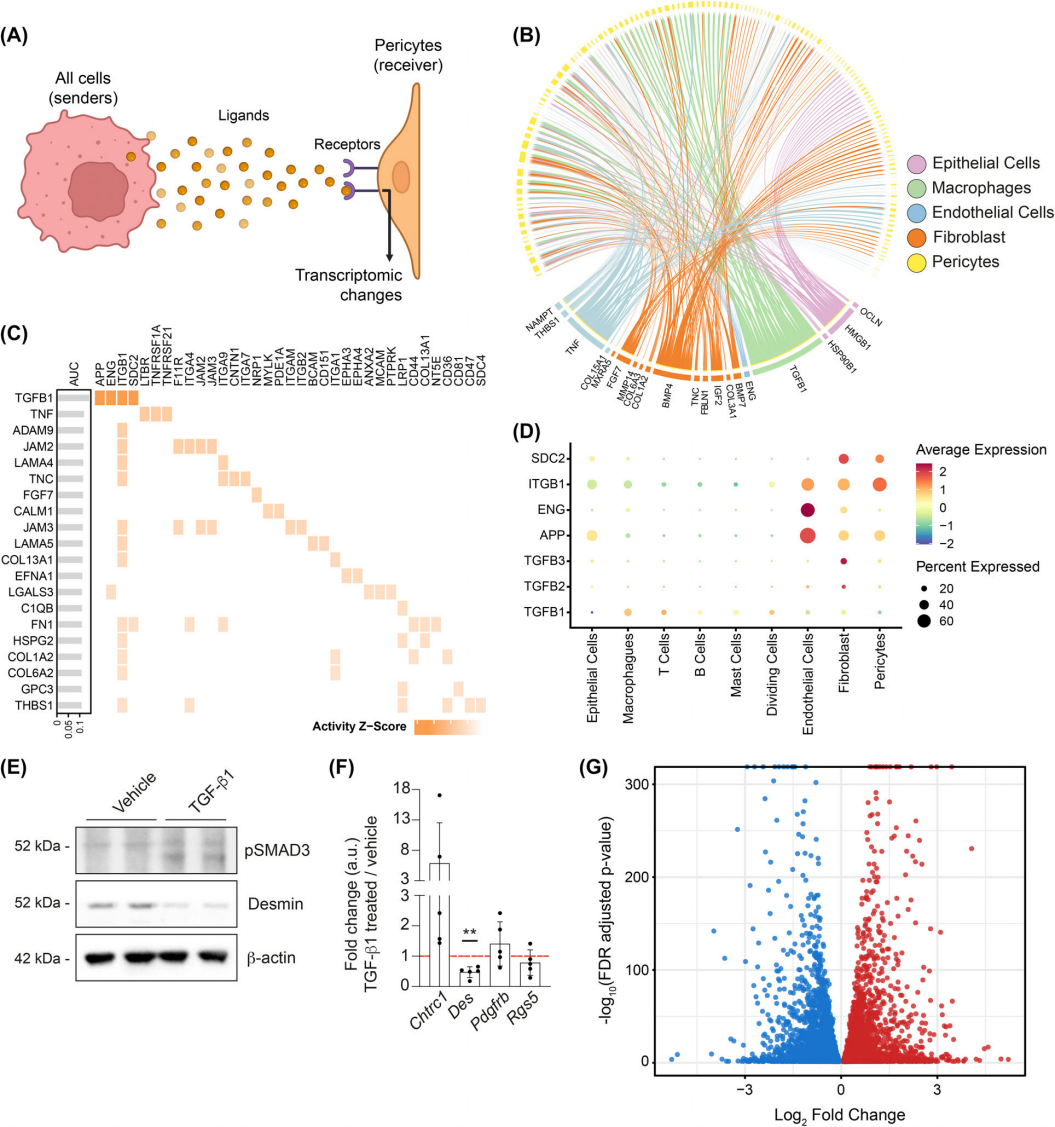
Pericyte dysfunction is orchestrated by TGF‐β. (A) The scheme illustrates our proposed hypothesis that pericyte dysfunction emanates from a paracrine signal. (B) Circle plot showing the potential ligand–receptor interaction among pericytes as the primary receivers and other (nonpericytes) prostate cell types as senders. The analysis identifies key ligands and signaling pathways involved in cellular communication. (C) The top ligand–receptor interactions between pericytes and the rest of prostate cell types predicted by NicheNet analysis. (D) Gene expression by cell type, with dot size indicating the percentage of cells expressing potential TGF‐β pericyte receptors and color representing expression level. (E) Representative western blot of brain pericytes treated with TGF‐β for 24 h and subjected to immunoblotting for phospho‐SMAD3 (p‐SMAD3) and Desmin. β‐Actin was used as a loading protein control. These experiments were done in three biological replicates. Quantifications are shown in Fig. S3C. (F) Relative gene expression of *Chtrc1*, *Des*, *Pdgfrb*, and *Rgs5* genes after treatment with TGF‐β1 and analyzed by quantitative polymerase chain reaction. Statistical analysis was performed by a one‐sample *t*‐test (*n* = 4–5). **P ≤ 0.01. Data represent mean ± SEM. Each dot corresponds to an individual biological replicate (*n* = 4). (G) Volcano Plot representing the total amount of DEG with an FDR‐adjusted *P*‐value > 0.5 and logFoldChange > 0 (red) and < 0 (blue). Each point represents a gene. Transcriptomic analysis was done in *n* = 4 per experimental condition.

Next, we hypothesized that mural cell dysfunction in prostate tumors would emanate from a paracrine signal. Thus, we applied Nichenet to our scRNAseq dataset [36]. We defined pericytes as receivers of the potential signal, and the rest of the cells as senders, focusing on ligand–receptor pairs showing differential gene expression between normal and tumor conditions within our receiver cell type (Fig. 3A). We identified a potential role for TGF‐β in the regulation of pericyte function based on three complementary pieces of evidence: (i) The cell communication analysis revealed a predominant TGF‐β‐based communication between tumor‐associated macrophages and pericytes (Fig. 3B); (ii) the top ligand predicted by the analysis that may be driving tumor‐associated pericyte phenotypes was TGF‐β (Fig. 3C), and (iii) TGF‐β‐associated transcriptional changes in pericytes were the most remarkable of all analyzed ligands (Fig. S3A). Analysis of TGF‐β receptor expression in pericytes identified a predominance of non‐canonical receptors (SDC2) and coreceptors (ITGB1) as potential mediators of signaling (Fig. 3D) [40, 41].

To provide causal evidence of the role of TGF‐β in pericyte function, we generated pericyte cultures derived from murine brain tissue and validated that these cells retained pericyte markers in culture (Fig. S3B). Next, we supplemented culture media with TGF‐β for 24 h and validated that TGF‐β induced downstream pathway activation by means of Smad3 phosphorylation (Ser423/425) (Fig. 3E and Fig. S3C) and mRNA expression of a canonical target of the pathway (*Cthrc1*, Fig. 3F). As predicted, the mRNA and protein expression of a functionality marker of pericytes (*Desmin*) was reduced upon TGF‐β treatment, whereas housekeeping pericyte markers (*Pdgfrb and Rgs5*) remained roughly unaltered (Fig. 3E,F). Importantly, the effect of TGF‐β on *Desmin* expression was partially rescued upon inhibition of SMAD3 using SIS3 [42] (Fig. S3D).

To unravel the biological and functional consequences of paracrine TGF‐β signaling in pericytes, we performed bulk RNA sequencing on cultured pericytes treated with TGF‐β or vehicle for 48 h. TGF‐β treatment elicited a robust transcriptional response, with 8487 differentially expressed genes (FDR adjusted *P*‐value < 0.05, Fig. 3G and Table S2). Interestingly, functional enrichment analysis revealed an alteration in contractility processes upon TGF‐β treatment (Fig. S3E). Of note, within the KEGG‐derived cell contractility signature, *Myh11* and *Acta2* caught our attention, as both have been associated with pericyte function [10]. By plotting the normalized expression counts of each gene, we could validate that their expression was downregulated in cultured pericytes upon treatment with TGF‐β (Fig. S3F).

**Fig. 4:**
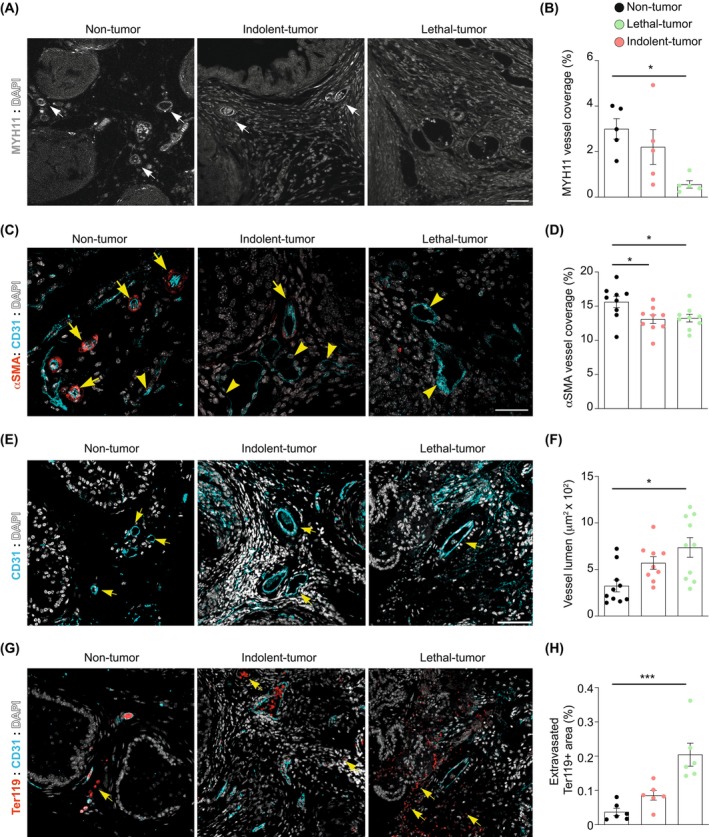
Pericyte dysfunction results in vessel leakage in prostate cancer. (A) Representative images of non‐tumor (Pb‐Cre‐PTEN^WT/WT^), indolent‐tumor (Pb‐Cre‐PTEN^flox/flox^), and lethal‐tumor (Pb‐Cre‐PTEN^flox/WT^; LKB1^flox/flox^) prostates stained for MYH11 (gray) and DAPI (white). Staining was performed in *n* = 5 mice per genotype. Scale bars, 50 μm. White arrows show positive MYH11 vessels. (B) Quantification of MYH11 vessel coverage. *n* = 5 mice per genotype. Data represent mean ± SEM. Each dot corresponds to an individual mouse. Statistical analysis was performed by Kruskal–Wallis test. *P ≤ 0.05. (C) Representative images of non‐tumor (Pb‐Cre‐PTEN^WT/WT^), indolent‐tumor (Pb‐Cre‐PTEN^flox/flox^), and lethal‐tumor (Pb‐Cre‐PTEN^flox/WT^; LKB1^flox/flox^) prostates stained for endothelial cells (CD31, cyan), pericytes (αSMA, red), and DAPI (white). Staining was performed in *n* = 9 mice per genotype. Scale bars, 25 μm. (D) Quantification of αSMA vessel coverage. *n* = 9 mice per genotype. Data represent mean ± SEM. Each dot corresponds to an individual mouse. Statistical analysis was performed by Kruskal–Wallis test. *P ≤ 0.05. (E) Representative images of non‐tumor (Pb‐Cre‐PTEN^WT/WT^), indolent‐tumor (Pb‐Cre‐PTEN^flox/flox^), and lethal‐tumor (Pb‐Cre‐PTEN^flox/WT^; LKB1^flox/flox^) prostates stained for endothelial cells (CD31, cyan) and DAPI (white). Staining was performed in *n* = 10 Pb‐Cre‐PTEN^WT/WT^ and Pb‐Cre‐PTEN^flox/WT^; LKB1^flox/flox^ mice, and in *n* = 9 Pb‐Cre‐PTEN^flox/flox^ mice. Scale bars, 50 μm. Arrows indicate vessels. (F) Quantification of vessel lumen. *n* = 10 Pb‐Cre‐PTEN^WT/WT^ and Pb‐Cre‐PTEN^flox/WT^; LKB1^flox/flox^ mice, and *n* = 9 Pb‐Cre‐PTEN^flox/flox^ mice. Data represent mean ± SEM. Each dot corresponds to an individual mouse. Statistical analysis was performed by Kruskal–Wallis test. *P ≤ 0.05. (G) Representative images of nontumor (Pb‐Cre‐PTEN^WT/WT^), indolent‐tumor (Pb‐Cre‐PTEN^flox/flox^), and lethal‐tumor (Pb‐Cre‐PTEN^flox/WT^; LKB1^flox/flox^) prostates stained for endothelial cells (CD31, cyan), erythrocytes (Ter119, red), and DAPI (white). Staining was performed in *n* = 6 mice per genotype. Scale bars, 100 μm. Arrows indicate Ter119 extravasation. (H) Quantification of Ter119 expression outside the blood vessels. *n* = 6 mice per genotype. Data represent mean ± SEM. Each dot corresponds to an individual mouse. Statistical analysis was performed by Kruskal–Wallis test. ***P ≤ 0.001.

Our results suggest that the presence of a prostate tumor and the production of TGF‐β could reprogram pericytes into a dysfunctional state that is associated with reduced expression of contractility markers. To validate these data, we assessed the expression of myosin chain 11 (MYH11) and α smooth muscle actin (αSMA) in tumor‐associated pericytes *in situ* [43]. Indeed, these markers exhibited a reduction in murine prostate tumors (Fig. 4A–D and Fig. S4A,B). Since the loss of pericyte contractility could influence vessel integrity, we assessed the changes in vessels within these tumor models. The pathogenesis and progression of prostate tumors were associated with an increased vascular lumen (Fig. 4E,F, and Fig. S4C) and erythrocyte extravasation, indicative of elevated vascular permeability (Fig. 4G,H, and Fig. S4D).

## 4 Discussion

The architecture and function of blood vessels are severely altered in cancer [11, 44]. The abundance and permeability of vessels are influenced by signals that regulate endothelial cell function [45]. In this regard, pericytes are critical components in the final layout and function of the vascular bed [10]. Although molecular changes in pericytes that contribute to functional alterations in tumors have been reported [11], the consensus in the field is that pericytes are lost across disease progression [9, 10, 46]. Our analysis of human and murine prostate cancer specimens demonstrates that an imbalance of pericytes and endothelial cells is strongly associated with disease progression. Yet, this imbalance is caused not only by a loss of pericyte numbers but also by their reprogramming into a cell state that exhibits reduced function. Our data are in line with recent observations showing that expansion and reprogramming of mesenchymal cells are early events in prostate cancer and that these changes are associated with disease progression [8].

The complexity and tissue specificity of pericytes have limited the definition of tissue‐agnostic pericyte markers, which has posed a limitation when studying the abundance and function of these cells in pathological conditions. With the advent of single‐cell molecular technologies, our capacity to categorize cell states and markers within cell types has dramatically increased [8, 10, 37, 47, 48, 49]. Here, we have leveraged bulk and single‐cell transcriptomics analyses to build gene expression scores that inform about changes in the balance of endothelial cells and pericytes. In addition, we have defined pericyte markers and differentiation indicators to build pericyte functional scores. This approach reveals that, beyond the reported loss of balance between endothelial cells and pericyte abundance in tumors [13, 16, 18, 50, 51], we can also identify alterations in the function of pericytes that are associated with the presence of an aggressive malignancy. With this new evidence, it is tempting to speculate that previous studies reporting pericyte loss in other cancerous settings could also present a component of pericyte dysfunction that has been overlooked. This is consistent with many scRNAseq data sets, in which many more mural cells are recovered in tumors compared to normal tissue. This is also in line with the notion that pericytes exhibit great cell plasticity [11, 47]. Our study proposes a novel strategy for evaluating pericyte behavior in tumors, utilizing scores that combine pericyte housekeeping markers and cell function indicators to faithfully reflect vascular phenotypes. Yet, it is important to note that these markers may vary in tumors arising from different tissue types and lineages.

An intriguing observation of our data is that the loss of functional properties in tumor pericytes is orchestrated by TGF‐β signaling. Elevated TGF‐β tone has been previously linked to aberrant stromal behavior and poor prognosis in cancer, primarily through a key role in CAF activation [20, 52] and in promoting endothelial cell growth [53]. Here, we extend the pleiotropic role of this cytokine, showing that TGF‐β also promotes pericyte dysfunction. This observation aligns with the role of this cytokine in regulating pericyte function in fibrosis [10, 54, 55]. We speculate that TGF‐β elicits a pericyte phenotype that reduces its regulatory activity in vessels, thus leading to increased vascular permeability. Whether the reprogramming of pericytes by TGF‐β activates new functions in pericytes remains to be studied.

Overall, our results revisit the contribution, function, and regulation of pericytes in prostate cancer and open new opportunities to understand the process of disease progression and dissemination.

## 5 Conclusions

In summary, we have identified that an imbalance between pericytes and endothelial cells is strongly associated with prostate cancer disease progression. Furthermore, we demonstrate that this imbalance is not only caused by a loss of pericyte numbers but also by their reprogramming into a cell state that exhibits reduced function. Finally, we report that functional reprogramming in prostate cancer is elicited by TGFβ signaling in pericytes.

## Conflict of interest

The authors declare no conflict of interest.

## Author contributions

M.G., A.C., A.M‐R., and A.M‐L. were the main contributors in the conception, design, acquisition, and interpretation of the data and in writing the article. A.M‐R., A.M‐L., S.G‐L., J.G‐B., H.v.S., I.A., A.E., L.B‐B., I.M., and P.V. performed experiments and data analysis with input from A.C. and M.G. Bulk RNA sequencing and scRNAseq analyses were performed by A.M‐L. and I.M.

## Ethics approval

All mouse experiments were carried out following the ethical guidelines established by the Biosafety and Welfare Committee at CIC bioGUNE and following the recommendations from AAALAC.

## Supporting information


**Fig. S1.** Dot plot showing marker expression across cell types in prostate tissue.
**Fig. S2.** Low‐magnification images corresponding to those shown in Fig. 2.
**Fig. S3.** Impact of TGF‐β signaling in pericytes.
**Fig. S4.** Low‐magnification images corresponding to those shown in Fig. 4.

**Table S1.** scRNAseq patient data.

**Table S2.** Differentially expressed genes in bulk RNA‐seq data of cultured pericytes.

## Data Availability

Transcriptomic data of endothelial (*PECAM1* and *CDH5*) and pericyte genes (*CD248, DES, NOTCH3, PDGFRB*, *and RGS5*) was assessed using the online bioinformatics platform Cancertool (https://cancertool.cicbiogune.es/CANCERTOOL/) [5]. Cancertool includes data of expression, disease‐free survival, and gene enrichment analysis based on seven different prostate cancer transcriptomic datasets (Glinsky *et al*., Donated by MSKCC; Grasso *et al*., GSE35988; Lapointe *et al*., GSE3933; Taylor *et al*., GSE21032; TGCA, Firehose Broad; Tomlins *et al*., GSE6099; Varambally *et al*., GSE3325). Patient‐derived bulk expression data can be obtained from the Gene Expression Omnibus (GEO, https://www.ncbi.nlm.nih.gov/geo/), and the selected studies with the detailed patient information are listed in Table S1. Previously published scRNA‐seq data reanalyzed here are available under accession codes: GSE141445 [30], GSE181294 [31], and GSE176031 [29]. RNAseq data on cultured pericytes is deposited in the BioProject repository (NCBI) (GSE278801). No new algorithms were developed for this article. All code generated for analysis is available from the authors upon request.
