# Supplementary material for: Aggressive prostate cancer is associated with pericyte dysfunction
Source: Mol Oncol. 2025 Oct 21;20(4):947–61. doi: 10.1002/1878-0261.70135 (PMC13060648; doi:10.1002/1878-0261.70135)
Supplement: Supplementary file 1 — Fig. S1. Dot plot showing marker expression across cell types in prostate tissue. Fig. S2. Low‐magnification images corresponding to those shown in Fig. 2. Fig. S3. Impact of TGF‐β signaling in pericytes. Fig. S4. Low‐magnification images corresponding to those shown in Fig. 4. [file MOL2-20-947-s001.pdf]

# Supplementary Figures

*Martinez-Romero, Martinez-Larrinaga* et al.

# Supplementary Figure 1

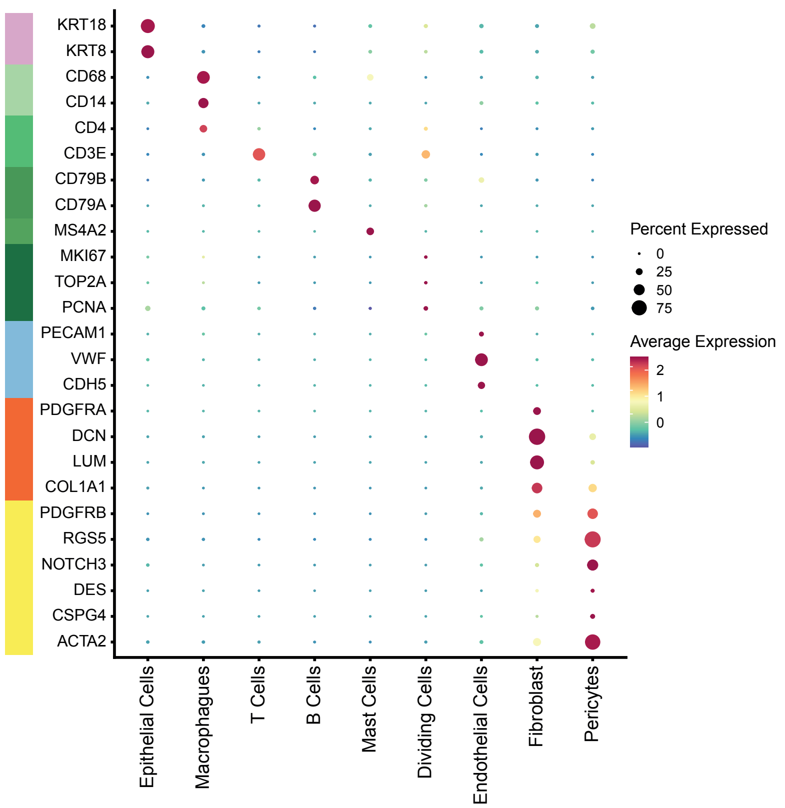

**Supplementary Figure 1. Dot plot showing marker expression across cell types in prostate tissue.** Marker gene expression across cell types, with dot size indicating the percentage of cells expressing each marker and color representing expression level.

Supplementary Figure 2

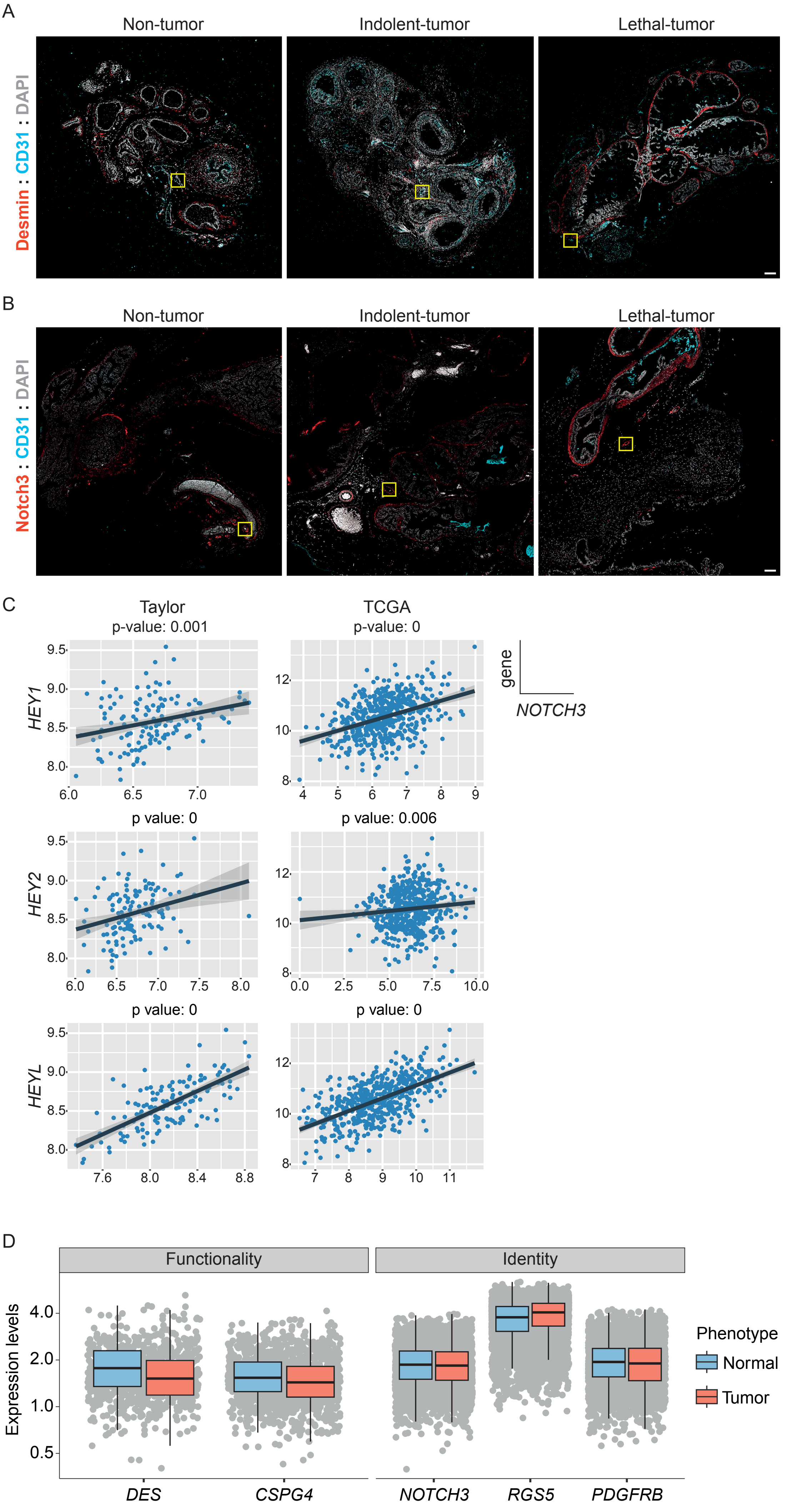

**Supplementary Figure 2. Low-magnification images corresponding to those shown in Figure 2.** (**A**) Low-magnification images of non-tumor (Pb-Cre-PTEN$^{WT/WT}$), indolent-tumor (Pb-Cre-PTEN$^{flox/flox}$), and lethal-tumor (Pb-Cre-PTEN$^{flox/WT}$; LKB1$^{flox/flox}$) prostates stained for endothelial cells (CD31, cyan), pericytes (Desmin, red), and DAPI (white). Saining was performed in *n* = 10 Pb-Cre-PTEN$^{WT/WT}$ and Pb-Cre-PTEN$^{flox/WT}$; LKB1$^{flox/flox}$ mice, and in n = 8 Pb-Cre-PTEN$^{flox/flox}$ mice. Scale bars, 100 μm. (**B**) Low-magnification images of non-tumor (Pb-Cre-PTEN$^{WT/WT}$), indolent-tumor (Pb-Cre-PTEN$^{flox/flox}$), and lethal-tumor (Pb-Cre-PTEN$^{flox/WT}$; LKB1$^{flox/flox}$) prostates stained for endothelial cells (CD31, cyan), pericytes (Notch3, red), and DAPI (white). Saining was performed in *n* = 10 mice per genotype. Scale bars, 100 μm. The yellow squares in **A** and **B** illustrate the corresponding high magnification images shown in **Fig. 2B, C**. (**C**) Scatter plots showing the Spearman correlation between *NOTCH3* and *HEY1*, *HEY2*, and *HEYL* expression in primary tumor samples from the Taylor and TCGA prostate cancer datasets. (**D**) Box plot representing the gene expression changes in the mural cell compartment across phenotypes (Normal ~ Tumor). Each point represents one cell. Individual values represent the total cell median for each group; the value between gene groups (Normal vs. Tumor) represents the difference between the medians.

# Supplementary Figure 3

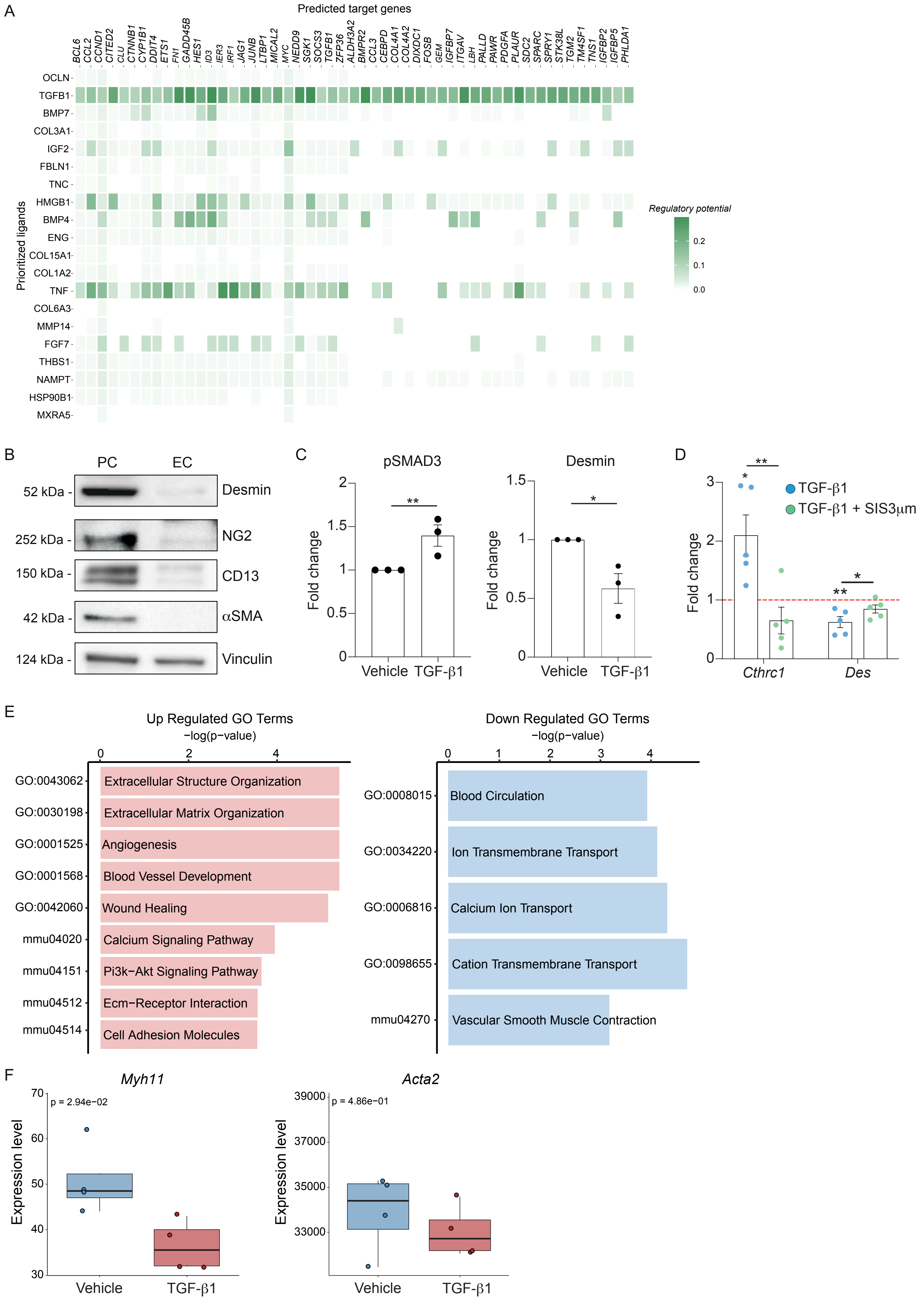

**Supplementary Figure 3. Impact of TGF-β signaling in pericytes**. (**A**) The predicted downstream target genes in tumor pericytes by NicheNet algorithm. (**B**) Wildtype brain pericytes (PC) and endothelial cells (EC) were subjected to immunoblotting for pericyte markers (Desmin, NG2, CD13, and αSMA). (**C**) Quantification of p-SMAD3 and Desmin expression levels normalized to β-actin. Graphs show the mean of three biological replicates per group. Each dot represents a biological replicate. A representative western blot is shown in **Fig. 3E**. Statistical analysis was performed by a one-sample t-test. *$P \leq 0.05$ and **$P \leq 0.01$. (**D**) Relative gene expression of *Des* after TGFβ treatment and Smad3 inhibitor (SIS3) analyzed by quantitative polymerase chain reaction. Graphs show the mean of five biological replicates per group. Each dot represents a biological replicate. Statistical analysis was performed by a two-way ANOVA test ($n = 5$). *$P \leq 0.05$ and **$P \leq 0.01$. (**E**) Gene Ontology (GO) analysis of the transcriptional programs upregulated and downregulated in wild-type brain pericytes treated with TGF-β1 for 48h. (**F**) Box plots showing the expression levels of *Myh11* (ENSMUSG00000018830) and *Acta2* (ENSMUSG00000035783) in bulk RNA-seq data shown in **Fig. 3G**. Each dot represents an individual biological replicate.

Supplementary Figure 4

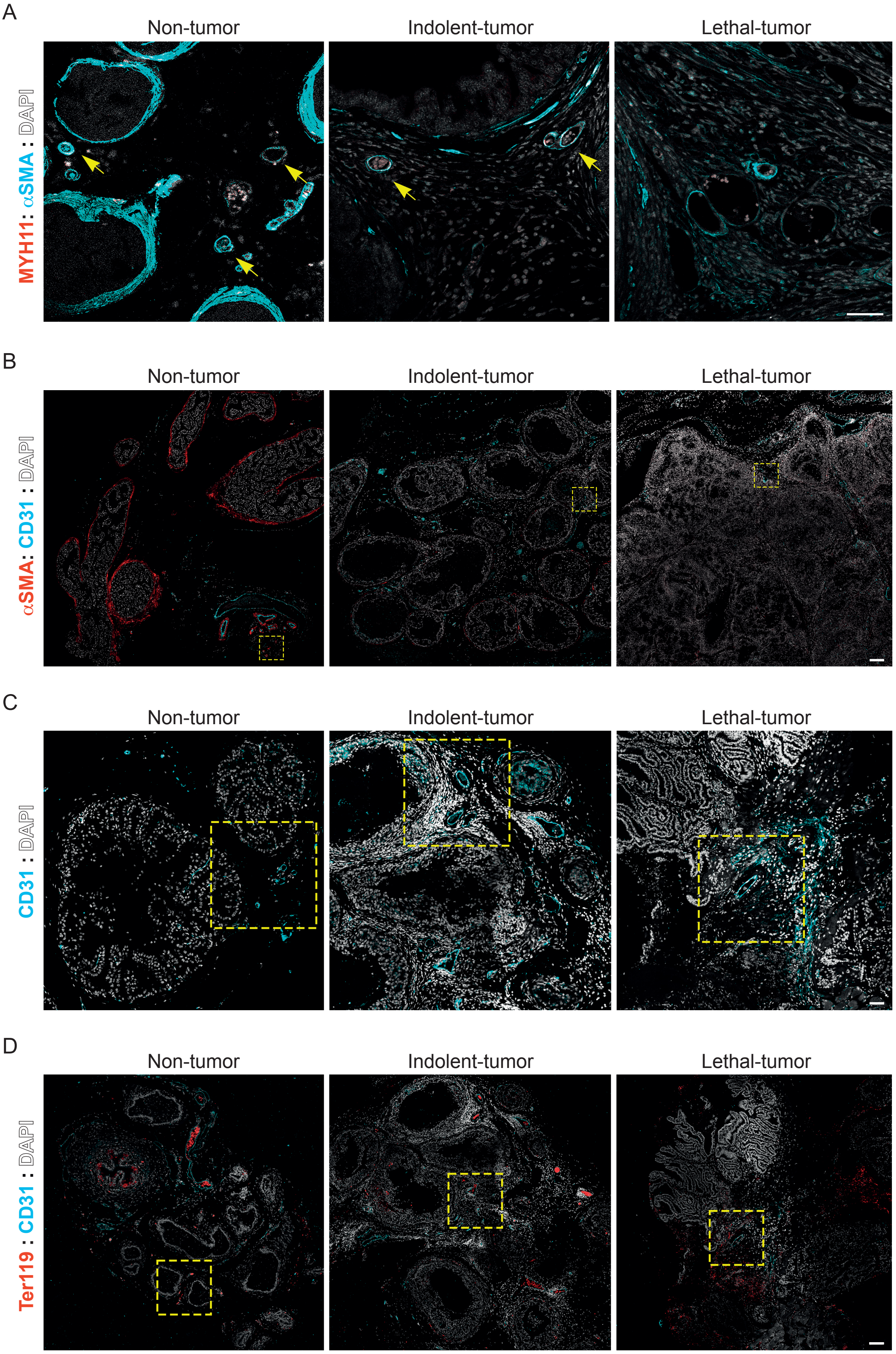

**Supplementary Figure 4. Low-magnification images corresponding to those shown in Figure 4.** (**A**) Representative images of non-tumor (Pb-Cre-PTEN$^{WT/WT}$), indolent-tumor (Pb-Cre-PTEN$^{flox/flox}$), and lethal-tumor (Pb-Cre-PTEN$^{flox/WT}$; LKB1$^{flox/flox}$) prostates stained for MYH11 (red), αSMA (cyan), and DAPI (white). Saining was performed in *n* = 5 mice per genotype. Scale bars, 50 μm. Yellow arrows show positive MYH11 vessels. (**B**) Low-magnification images of non-tumor (Pb-Cre-PTEN$^{WT/WT}$), indolent-tumor (Pb-Cre-PTEN$^{flox/flox}$), and lethal-tumor (Pb-Cre-PTEN$^{flox/WT}$; LKB1$^{flox/flox}$) prostates stained for endothelial cells (CD31, cyan), pericytes (αSMA, red), and DAPI (white). Saining was performed in *n* = 9 mice per genotype. Scale bars, 100 μm. (**C**) Low-magnification images of non-tumor (Pb-Cre-PTEN$^{WT/WT}$), indolent-tumor (Pb-Cre-PTEN$^{flox/flox}$), and lethal-tumor (Pb-Cre-PTEN$^{flox/WT}$; LKB1$^{flox/flox}$) prostates stained for endothelial cells (CD31, cyan) and DAPI (white). Saining was performed in *n* = 10 Pb-Cre-PTEN$^{WT/WT}$ and Pb-Cre-PTEN$^{flox/WT}$; LKB1$^{flox/flox}$ mice, and in n = 9 Pb-Cre-PTEN$^{flox/flox}$ mice. Scale bars, 50 μm. (**D**) Low-magnification representative images of non-tumor (Pb-Cre-PTEN$^{WT/WT}$), indolent-tumor (Pb-Cre-PTEN$^{flox/flox}$), and lethal-tumor (Pb-Cre-PTEN$^{flox/WT}$; LKB1$^{flox/flox}$) prostates stained for endothelial cells (CD31, cyan), erythrocytes (Ter119, red), and DAPI (white). Saining was performed in *n* = 6 mice per genotype. Scale bars, 100 μm. The yellow squares in **B**, **C**, and **D** illustrate the corresponding high magnification images shown in **Fig. 4**.**C**, **E**, **G**.
